# Supplementary material for: Content-rich biological network constructed by mining PubMed abstracts
Source: BMC Bioinformatics. 2004 Oct 8;5:147. doi: 10.1186/1471-2105-5-147 (PMC528731; doi:10.1186/1471-2105-5-147)
Supplement: Additional File 2 — The original results of the above study (non-essential files are deleted to keep the file size under the limit set by BMC bioinformatics). [file 1471-2105-5-147-S2.bz2 › chilibotAdditionalFile2/dip05/640ID7939681E11027/html/FCCB_FCCA.html]

 


 **FCCB** and **FCCA** 
  
Found 9 abstracts in PubMed, retrieved 05.  
 

 What does Google say? 
 PDF only 
| .edu only 

---

**Interactive relationship** (e.g. stimulation, inhibition, etc)

**Inhibitory relationship**- The results suggest that the reduction of methacrylate or acrylate by benzyl viologen radical is most likely catalyzed either by the periplasmic flavoprotein  **FccA**  or by a complex consisting of  **FccA**  and the predicted c type cytochrome  **FccB** .  Ref: 11685377 Arch Microbiol, 2001
**Neutral relationship**

**Non-interactive relationship** (e.g. studied together, co-existance, homology, etc.)

- 4 We have established that the constitutively expressed FCSD  [ **FCCA** ]   [ **FCCB** ]  genes soxEF1 are located elsewhere in the genome.  Ref: 11863431 Biochemistry, 2002
- by the Family and Child Services Department FCSD  [ **FCCA** ]   [ **FCCB** ] .  Ref: 12041067 Dom Law Rep< MedlineTA>Dom Law Rep, 2002
- In order to protect the unborn baby s health, the Superintendent of the FCSD  [ **FCCA** ]   [ **FCCB** ]  ordered its apprehension.  Ref: 12041067 Dom Law Rep< MedlineTA>Dom Law Rep, 2002
- Efforts to produce the holoproteins of the soluble Allochromatium vinosum FCSD  [ **FCCA** ]   [ **FCCB** ]  and the membrane bound Ectothiorhodospira vacuolata protein in Escherichia coli using several expression systems were unsuccessful.  Ref: 11479699 Arch Microbiol, 2001
- SoxF was 37% identical to the flavoprotein  **FccB**  of flavocytochrome c  [ **FCCA** ]  sulfide dehydrogenase of Allochromatium vinosum.  Ref: 11443084 J Bacteriol, 2001
- FCSD  [ **FCCA** ]   [ **FCCB** ]  is a heterodimer containing two different cofactors, a flavin FAD and one or two heme c groups, located on the separate protein subunits.  Ref: 11479699 Arch Microbiol, 2001
- Flavocytochrome c  [ **FCCA** ]  sulfide dehydrogenase FCSD  [ **FCCA** ]   [ **FCCB** ] , an enzyme that catalyzes the reversible conversion of sulfide to elemental sulfur in vitro, is common to bacteria that utilize reduced sulfur compounds as electron donors in the process of carbon dioxide fixation.  Ref: 11479699 Arch Microbiol, 2001
- Both Rhodobacter hosts were able to transcribe the genes coding for FCSD  [ **FCCA** ]   [ **FCCB** ]  from the rbcA promoter and to produce detectable amounts of recombinant FCSD  [ **FCCA** ]   [ **FCCB** ]  holoprotein.  Ref: 11479699 Arch Microbiol, 2001
- However, soluble cytochrome c 551 and flavocytochrome c  [ **FCCA** ]  FCSD  [ **FCCA** ]   [ **FCCB** ]  have previously been implicated in the oxidation of thiosulfate and sulfide on the basis of enzyme assays in Chlorobium.  Ref: 11863431 Biochemistry, 2002
- The Supreme Court found that a fetus was not subject to apprehension within the meaning of the legislation under which the FCSD  [ **FCCA** ]   [ **FCCB** ]  acted.  Ref: 12041067 Dom Law Rep< MedlineTA>Dom Law Rep, 2002
